# Care and Complexity in Emergency Housing: an Examination of the COVID-19 Shelter-in-Place (SIP) Hotel Program to House People Experiencing Homelessness in San Francisco

**DOI:** 10.1007/s11524-022-00705-8

**Published:** 2023-01-18

**Authors:** Elizabeth Abbs, Naomi Schoenfeld, Mason Lai, Shannon Satterwhite, Sara Zhou, Joshua Bamberger, Barry Zevin

**Affiliations:** 1 San Francisco Department of Public Health, San Francisco, CA USA; 2 University of California, San Francisco, San Francisco, CA USA; 3 UC Davis Health, Sacramento, CA USA; 4 Veteran’s Administration Medical Center, San Francisco, CA USA

**Keywords:** Homelessness, COVID-19, Housing First, Socioeconomic disparity

## Abstract

In this study, we consider the patient, provider, and public health repercussions of San Francisco’s (SF) COVID-related response to homelessness using tourist hotels to house people experiencing homelessness (PEH). We describe the demographics, medical comorbidities, and healthcare utilization patterns of a subset of PEH who accessed the shelter-in-place (SIP) hotel sites during the 2020–2021 pandemic. We focus on how SIP hotels impacted connection to outpatient care and higher-cost emergency utilization. Our mixed methods study integrates qualitative and quantitative data to consider the impact of this temporary housing initiative among a medically complex cohort in a time of increased morbidity and mortality related to substance use. We found that temporary SIP housing increased outpatient care and reduced higher-cost hospital utilization. Our results can inform the future design and implementation of integrated supportive housing models to reduce mortality and promote wellness for PEH.

## Introduction

People experiencing homelessness (PEH) routinely calculate risk and value to avoid serious bodily and/or psychological harm to meet immediate needs for survival. These calculations often take precedence over longer-term needs including medical or psychiatric care [1, 2]. Survival can involve bargaining one harm in order to mitigate another, leading to disproportionately high rates of all-cause morbidity and mortality [3, 4]. In March 2020, PEH were faced with yet another layer of risk: exposure to the COVID-19 virus [5].

**Fig. 1:**
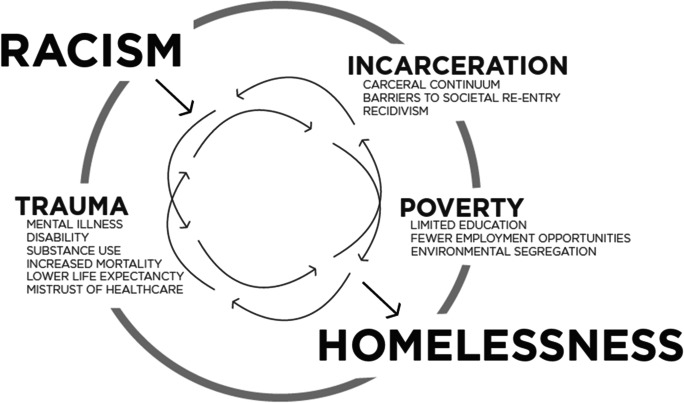
Cycle of injustice(s) central to racial disparity in homelessness

PEH face multiple, overlapping forms of stigmatization and oppression [6, 7]. Housing insecurity delays healthcare [1], disproportionately impacting communities of color and compounding disparities. People of color make up the largest share of PEH, with Black and African American people representing 40% of PEH while making up only about 5% of the overall population of San Francisco (SF) [8]. As depicted in Fig. 1, overt and structural racism are significant factors that directly and indirectly impact higher rates of incarceration, trauma, and lost economic opportunities [8–11]. Further, high-cost healthcare utilization is independently associated with incarceration [12]. As such, the social and historical impact of structural racism in SF affects the health and wellness of PEH and directly impacts a Black individual’s experience of homelessness and their relationship with public services, including housing and healthcare.

In the USA, SF was among the first cities to institute a shelter-in-place (SIP) ordinance on March 16, 2020, garnering national headlines as a model of “aggressive public health.” The COVID-19 pandemic was predicted to have a devastating effect on PEH due to inadequate access to hygiene and sanitation and expected impediments to care delivery for a population with high prevalence of underlying medical conditions [13, 14]. Advocates for PEH raised the alarm and applied political pressure [15]. In April 2020, an outbreak at SF’s largest homeless shelter impacting 67% of the residents and 17% of the staff prompted a rapid decision to lease 7000 hotel rooms with medical and social support staff for a homeless population estimated just over 8000 [15, 16]. This intervention created an unprecedented opportunity to better understand the impact of temporary supportive hotel housing on PEH. In this article, we draw from quantitative and qualitative data to examine the SIP hotel intervention, both the value and limitations of the program. This study adds to the limited available data on temporary housing, including its social, medical, and psychological impact.

### Background

While permanent housing is the ideal solution to homelessness and has demonstrated benefit, temporary housing has been shown to increase primary care utilization in vulnerable populations of PEH [17–19]. Our study aims to describe the clinical and demographic makeup of residents accessing SIPs in SF.

The SF COVID-19 SIP hotel program was funded by the Federal Emergency Management Agency (FEMA), the CARES Act, and the San Francisco General Fund [20, 21]. PEH came to SIP through four general routes via (1) direct transfer from congregate shelter after the April 2020 outbreak; (2) hospital discharges; (3) outreach targeting PEH meeting COVID vulnerability criteria; and (4) additional vulnerability criteria developed by the leadership of San Francisco Department of Public Health (SFDPH) Street Medicine and Shelter Health taking into consideration race, ethnicity, and gender identity. Commissioned hotels and motels provided a room, private bathroom, three daily meals, and onsite clinical care and social services. As of July 2021, SF provided temporary housing to 3877 [21, 22] in private hotels, motels, and trailers, with nearly 1700 additional PEH living in congregate shelters and safe sleeping sites.

The SIP initiative provided more comprehensive services and staffing than other temporary congregate shelters or existing permanent supportive housing. SIPs were managed by community-based organizations (CBOs) with experience providing services for PEH. High rates of overdose deaths in the program’s first month led to the addition of robust harm reduction services including wellness checks, naloxone, supplies for safe drug consumption, and prescribed managed alcohol for select residents. All SIP hotels evolved to have nursing with clinical coverage (MD, NP, PA) at least a half day per week and as needed consultation from behavioral health clinicians and occupational and physical therapists.

## Methods

### Study Sample

For our quantitative portion, we included all people at least 18 years old who lived in a SIP hotel site for a minimum of 3 months. We excluded participants living in non-hotel SIP sites, such as shelters, tent encampments, recreational vehicles (RVs), sobering centers, medical respite, or other pre-COVID-19 navigation centers. For our qualitative portion, we included SIP staff available to attend a 90-min recorded research call to debrief their experience. The institutional review board of the University of California, San Francisco (UCSF) approved this study.

### Data and Measures

**Fig. 2:**
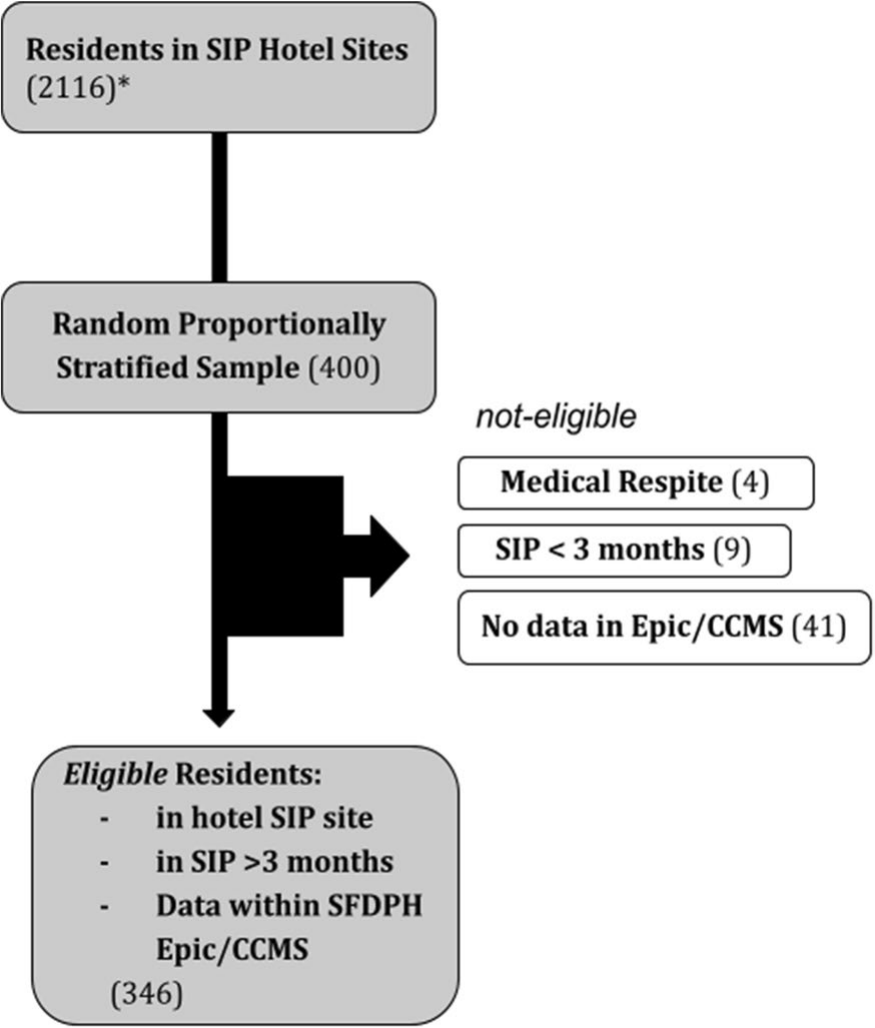
Quantitative sample selection

We conducted a cross-sectional analysis of residents staying in SIP hotels. We used the October 2020 SIP census from SFDPH’s Complex Care Management System (CCMS, a composite database of medical-psycho-social data) (*N* = 2116) to select a random sample of 400 participants, proportionally stratified by size of the SIP hotel. Of the 400 selected participants, 54 (14%) were ineligible: 41 with no record within Epic (the electronic medical record for SFDPH) or the CCMS database, nine with inadequate stay in SIP, and four with placement within a medical respite site (Fig. 2). Our team created a data dictionary and a standardized coding procedure. To assess reliability, different investigators coded five duplicate records that revealed a high inter-rater reliability with an unweighted Cohen’s kappa score of 0.85. We compared emergency department (ED) usage among SIP residents to monthly census data from the San Francisco General Hospital (SFGH), the city’s county hospital. We estimated annualized rates of inpatient and ED visits by doubling our 6-month data. Our focus group was based on a series of questions around perceived strengths, weaknesses, sustainability, and lessons from the SIP project. We transcribed the virtual session then performed analysis in grounded theory to underline shared perceptions.

### Healthcare Utilization, Cost, Engagement, and Retention in Care

Our primary outcomes included healthcare utilization patterns following SIP housing. To determine higher-cost healthcare utilization, we compared the number of ED and hospital admissions in the SFDPH Epic medical record 6 months prior to SIP housing to the number of ED visits and hospital admissions 6 months after SIP. We defined “acute care utilizers” as individuals with any ED or hospital utilization 6 months prior and/or after SIP hotel entry, and “non-acute care utilizers” as individuals with zero hospital touchpoints in Epic. To determine lower-cost healthcare utilization and retention in care, we assessed the presence of outpatient encounters 6 months prior to SIP and compared it to the presence of encounters 6 months after SIP. We counted on-site medical and nursing care as an outpatient encounter when a note was left in Epic; we counted clinician and other staff (nursing, social work, behavioral health, physical therapy) encounters separately.

### Sociodemographics, Health Status, and Substance Use

We assessed age, gender (assuming cis-gender when not otherwise indicated), ethnicity, race, and preferred language. Using CCMS data, we explored number of years homeless, history of incarceration, and utilization of other healthcare services (urgent care, psychiatric emergency, behavioral health, sobering center, medical respite). We defined connection to primary care as being assigned a primary care doctor in Epic. We determined the source of SIP referral by Epic documentation of housing prior to SIP; when not visible, we categorized type of homelessness as “unknown.”

We used the Elixhauser Index as a metric of medical complexity [23]. We counted the number of active medications in residents’ Epic chart to depict polypharmacy and medical complexity among patients engaged in care. We evaluated prevalence of mental health disorders, categorizing chart diagnosis by anxiety, depression, psychosis (either primary or drug-induced), post-traumatic stress disorder (PTSD), personality disorder, and other.

We evaluated substance use by chart diagnosis of alcohol, tobacco, opioid, stimulant, and benzodiazepine use (including ICD-10 codes, provider notes, and behavioral health screenings). To document access to addiction specialty care, we measured the prevalence of addiction medicine consultation notes. We counted medications for opioid use disorder (MOUD) such as methadone, buprenorphine, and naltrexone prior to and after SIP housing.

### Analytic Strategy

We collected and managed data using REDCap and used StataMP 16.1 for analysis with chi squared and Student’s *t* tests to evaluate differences between utilization groups. We used non-parametric Wilcoxon matched pairs signed-rank test to compare distributions of ED visits and inpatient encounters before and after SIP placement and McNemar’s test to determine differences in outpatient care utilization before and after SIP.

## Results

Table 1 depicts sociodemographic characteristics of our sample stratified by “acute care utilizer” versus “non-utilizer,” as defined above. We incorporated quotations and themes from our qualitative analysis to reinforce themes of medical complexity, lower-cost healthcare utilization, and complications of substance use.

**Table 1:** Characteristics of homeless adults with and without change in emergency department (ED) utilization after housing in shelter-in-place (SIP)

Characteristics	Total (*N* = 346)	Non-acute care utilizers (*N* = 148)	Acute care utilizers (*N* = 198)	*P* values
Individual risk factors				
Age, years median (interquartile range)	54.0 (42.0, 63.0)	54.5 (42, 62.5)	53.5 (42.0, 63)	0.80
Men, *N* (%)	237 (68.5)	91 (61.5)	146 (73.7)	0.02
*Gender identity*				
Cis male	212 (64.4)	81 (58.3)	131 (69.0)	0.05
Cis female	104 (31.6)	55 (39.6)	49 (25.8)	
Trans male/man	1 (0.30)	0 (0.0)	1 (0.5)	
Trans female/woman	9 (2.7)	3 (2.2)	6 (3.2)	
Gender nonbinary	3 (0.91)	0 (0.0)	3(1.6)	
*Ethnicity, N (%)*				
Hispanic	49 (14.2)	16 (10.8)	33 (16.7)	0.30
Non-Hispanic	295 (85.3)	131 (88.5)	164 (83.8)	
Unknown	2 (0.6)	1 (0.7)	1 (0.5)	
Race, *N* (%)				
African American	149 (43.0)	65 (43.9)	84 (42.4)	0.11
White	122 (35.3)	58 (39.2)	64 (32.3)	
Asian	19 (5.5)	9 (6.1)	10 (5.1)	
American Indian/Alaska Native	3 (0.9)	0 (0.0)	3 (1.5)	
Native Hawaiian/Pacific Islander	4 (1.2)	2 (1.4)	2 (1.0)	
More than one race	24 (6.9)	4 (2.7)	20 (10.1)	
Unknown	25 (7.2)	10 (6.8)	15 (7.6)	
*Preferred language*				
English	301 (89.9)	126 (90.0)	175 (89.7)	0.62
Spanish	24 (7.2)	10 (7.2)	14 (7.2)	
Chinese	2 (0.60)	0 (0.0)	2 (1.0)	
Vietnamese	1 (0.30)	0 (0.0)	1 (0.5)	
Russian	3 (0.90)	2 (1.4)	1 (0.5)	
Tagalog	1 (0.30)	1 (0.7)	0 (0.0)	
Other	3 (0.90)	1 (0.7)	2 (1.0)	
Environmental risk				
SIP within SOMA/Tenderloin	80 (23.1)	29 (19.6)	1 (25.8)	0.18
SIP outside SOMA/Tenderloin	266 (76.9)	119 (80.4)	147 (74.2)	
Number of years experiencing homelessness (years, IQR)*	10.0 (2, 18)	8.5 (2, 18)	10 (3, 18)	0.62
History of incarceration	184 (53.2)	79 (53.4)	105 (53.0)	0.95
*Housing location prior to SIP*				
Street/vehicle/makeshift shelter	108 (31.6)	41 (28.1)	67 (34.2)	0.01
Shelter/navigation center	72 (21.0)	24 (16.4)	48 (24.5)	
Institution (hospital, nursing facility, medical respite)	18 (5.3)	3 (2.1)	15 (7.7)	
Housed independently	11 (3.2)	5 (3.4)	6 (3.1)	
Other	4 (1.2)	2 (1.4)	2 (1.0)	
Unknown	129 (37.7)	71 (48.6)	58 (29.6)	
Service utilization				
Had primary care provider	200 (58.5)	75 (51.4)	125 (63.8)	0.02
Service use				
Sobering center	34 (9.8)	11 (7.43)	23 (11.62)	0.20
Medical respite	53 (15.3)	16 (10.8)	37 (18.7)	0.04
Psychiatric emergency services	80 (23.1)	23 (15.5)	57 (28.8)	< 0.01
Behavioral health/mental health (outpatient)	162 (46.8)	61 (41.2)	101 (51.0)	0.07
Jail	182 (52.6)	78 (52.7)	104 (52.5)	0.97
Urgent care**	255 (73.7)	100 (67.6)	155 (78.3)	0.03
Substance use				
Alcohol use	95 (27.5)	34 (23.0)	61 (30.8)	0.101
Tobacco use	154 (44.5)	57 (38.5)	97 (49.0)	0.05
Opioid use	98 (28.3)	38 (25.7)	60 (30.3)	0.34
Stimulant use	126 (36.4)	35 (23.7)	91 (46.0)	< 0.01
Benzodiazepine use	14 (4.05)	4 (2.7)	10 (5.1)	0.27
Other use	19 (5.5)	10 (6.8)	9 (4.6)	0.37
Nonfatal overdose	22 (6.4)	3 (2.0)	19 (9.6)	< 0.01
*Medications for substance use prior to SIP*				
Methadone	29 (8.4)	11 (7.4)	18 (9.1)	0.58
Buprenorphine	15 (4.3)	9 (6.1)	6 (3.0)	0.17
Naltrexone	2 (0.58)	1 (0.68)	1 (0.51)	0.84
*Medications for substance use after SIP*				
Methadone	22 (6.4)	8 (5.4)	16 (7.1)	0.53
Buprenorphine	31 (9.0)	16 (10.8)	15 (7.6)	0.30
Naltrexone	6 (1.7)	1 (0.7)	5 (2.5)	0.19
Addiction medicine consult encounter (inpatient/outpatient)	59 (17.9)	18 (12.3)	41 (22.3)	0.02
Mental health				
Anxiety	74 (21.4)	28 (18.9)	46 (23.2)	0.33
Depression	154 (44.5)	55 (37.2)	99 (50.0)	0.02
Psychosis NOS (schizophrenia/Schizoaffective disorder)	94 (27.2)	29 (18.9)	66 (33.3)	< 0.01
PTSD	44 (12.7)	15 (10.1)	29 (14.7)	0.21
Personality disorder	3 (0.9)	0 (0.0)	3 (1.52)	0.13
Other	19 (5.5)	6 (4.1)	13 (6.7)	0.31
Health status				
Elixhauser score	3 (1, 4)	2 (1, 3)	3 (2, 5)	< 0.01
Number of medications	4 (1, 7)	2 (0, 5)	5 (2, 9)	< 0.01
*Top 5 Elixhauser comorbidities*				
Drug use	179 (51.7)	64 (43.2)	115 (58.1)	< 0.01
Depression	139 (40.2)	48 (32.4)	91 (46.0)	0.01
Hypertension	113 (32.7)	38 (25.7)	75 (37.9)	0.02
Psychosis	90 (26.0)	29 (19.6)	61 (30.8)	0.02
Alcohol use disorder	82 (23.7)	31 (21.0)	51 (25.8)	0.30

^*^CCMS data only dates as far back as 2004

^**^Street medicine urgent care operates as primary care for some PEH

### Demographics

Of the 346 participants in our quantitative sample, majority were male (69%), non-LatinX (85%), English-speaking (90%), and with the largest racial group identified as Black (43%). Median age was 54 (IQR 42–63), and median experience of homelessness within SF was 10 years (range 0–24). The majority of residents lived on the street or in a vehicle (51%) or stayed in a congregate shelter (34%) prior to SIP.

Five staff attended the focus group; all were female with range of experience working in SIP from 6 weeks to 10 months. They had varied roles from onsite clinical staff to leadership to referrals; four of five attendees had prior experience working in shelter health supporting PEH.

### Medical Complexity

Forty-one participants (10%) with a profile in both Epic and CCMS were excluded due to having no medical encounters in Epic. Of the 346 quantitative participants with documented medical record, the most prevalent diagnoses were substance use disorder (58%), depression (47%), hypertension (37%), psychosis (30%), and alcohol use disorder (27%). Our qualitative data from SIP participant interviews, described elsewhere, highlighted the presence of two types of residents: those who engage in care and those who do not. Those who do not engage in care may be as sick/complex as those who do, but are harder to study through medical record. Our focus group of nurses highlighted the theme of risk and complexity behind closed doors.

Nurse participants in the focus group described how patients were often more complex than described in their medical record. One nurse who assisted with referrals to SIP shared, “we had to ask for people to be honest in their referrals. We’d get them there and they couldn’t get out of bed at all or couldn’t get out their wheelchair. It’d be a huge discrepancy when they came out to shelter health it was vastly different between what was reported and what they could actually pull off.”

Another nurse reflected,

whatever is on paper is different to what you see in person. Daily we see people who are struggling in shelter or navigation …there’s no place ...in the sky…even medical respite…we just navigate the best we can...let them sink or swim and document when they sink…there’s nowhere else for them to go besides the street because they’ve burned bridges or they are too sick or not sick enough...whether there’s criteria or not here’s very few people who fit.

These nurses stressed that SIP hotels could be, for some, unsafe and inappropriate given the high medical complexity. Yet, the SIP hotels were also often the best available option.

### System Utilization

Our data reveal patterns of system utilization corresponding with increased engagement in outpatient care and decreased use of ED facilities following SIP entry. The majority (58%) of our quantitative sample had a primary care provider (PCP) assigned in Epic. While 23 (7%) had no care utilization in SF prior to SIP, many had previously accessed various levels of care, including urgent care (78%), jail (56%), behavioral/mental health (50%), psychiatric emergency (25%), outpatient mental health crisis (18%), medical respite (16%), and sobering (11%).

### Hospital-Based Care (High-Cost System Utilization)

We stratified our sample by inpatient and emergency department utilization (Table 1), finding that “acute care utilizers” were more likely to be men (*p* = 0.015), have resided on the street prior to SIP entry (*p* = 0.005), and have a documented PCP (*p* = 0.021) with one or more outpatient clinician visit in the 6 months prior to SIP entry (*p* < 0.001). Acute care utilizers were also more likely to have higher medical-psychological complexity with higher rates of depression (*p* = 0.017), psychosis (*p* = 0.003), stimulant use (*p* < 0.001) with more prescribed medications (*p* < 0.001), and higher Elixhauser comorbidity indices (*p* < 0.001).

**Fig. 3:**
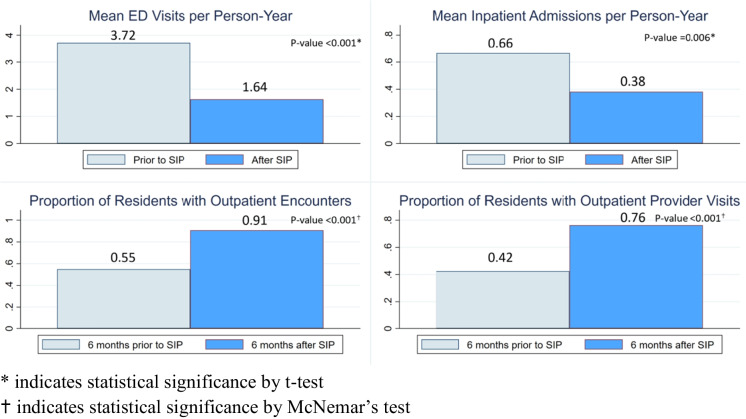
Emergency department (ED) and outpatient care utilization prior to and after shelter-in-place (SIP) hotel placement. Asterisk indicates statistical significance by *t* test. Dagger symbol indicates statistical significance by McNemar’s test

Figure 3 depicts ED utilization per person-year and outpatient care utilization 6 months prior to and 6 months after SIP. In the 6 months prior to SIP entry, 644 total ED visits were documented by the cohort, while only 284 total ED visits were documented in the 6 months after SIP entry. The mean number of ED visits decreased from 3.72 to 1.64 after SIP entry (*p* < 0.001, per person-year); the median number of ED visits remained at zero prior to and after SIP, although their distributions differed significantly (*p* < 0.001). Similarly, the mean number of inpatient encounters reduced from 0.66 to 0.38 after SIP entry (*p* < 0.001, per person-year); the median number of inpatient admissions remained at zero prior to and after SIP, although the distributions differed significantly (*p* < 0.001). In a sensitivity analysis, we compared the mean decrease in ED visits among our SIP subset to the SFGH’s overall change in ED census over the same time period. Our sample showed a mean change of – 1.05 ED visits per individual after SIP placement (*p* < 0.001), exceeding the expected change of – 0.30 ED visits per individual based on estimated secular trends over the course of 2020.

### Hotel and Clinic-Based Care (Lower-Cost System Utilization)

Active engagement with outpatient and preventative care can be difficult in homelessness due to a myriad of barriers, trauma, competing needs, and distrust in the medical system. The healthcare workers emphasized building relationships with SIP residents and the underlying nuances and complexity of providing care within temporary housing. They noted that some SIP residents readily accepted hotel-based medical care while others took longer to develop trust.

The focus group described how “trial and error” led to extensive “collaboration” with on and off-site CBOs and programs, adjusting staff and types of services offered to SIP residents, especially those who used substances. One nurse shared, “it takes time to build that trust; especially due to systemic trauma…This housing program was so rapid we didn’t have that trust and rapport.” The nursing staff also reflected on the expectation for nursing to provide medication management and transportation, two costly initiatives that were rarely feasible within SIP staff ratios or supported by city funding. Further, they expressed frustration and pushback when attempting to implement nursing stations in each SIP hotel. One nurse reflected, “I spent a good part of my money getting supplies because we had one blood pressure cuff and one thermometer for 400 people.”

Despite these difficulties, the prevalence of an outpatient visits after SIP increased significantly with clinician visits increasing from 42 to 79% (*p* < 0.001) and other staff visits increasing from 55 to 91% (*p* < 0.001) (Fig. 3).

### Substance Use Patterns

The pandemic changed patterns of substance use, disrupting the drug supply and access to medications for opiate use disorder (MOUD) in many states and hindering practices of harm reduction (such as not using alone) [24, 25]. The SIP initiative similarly altered the settings in which substance use occurs but quickly fostered an innovative approach to MOUD via buprenorphine delivery [26]. Further, many of SF’s methadone clinics were able to provide more take-home doses than prior to COVID. While the prevalence of OUD and interest in MOUD in the SIP hotels is poorly characterized in Epic, within our quantitative sample, 18% had Addiction Medicine specialty care visits and 28% had chart documentation of non-medical opioid (fentanyl or heroin) use. During this time period, buprenorphine prescriptions increased from 15 (4%) to 31 (9%) after SIP housing without statistical significance. In our cohort, concurrent use of multiple substances was common with 45% noted to use tobacco, 36% stimulants (methamphetamine or cocaine), 28% alcohol, and 4% benzodiazepines. Overdoses were also common; 6.4% had a non-fatal overdose recorded in Epic. Overdose deaths in SIP did occur, however none within our quantitative sample.

The common experience of overdose deaths in the first weeks of the program was devastating for the nursing staff. One nurse shared:

it was kind of like bittersweet. Clients, they finally got, I hate using the word, “housed,” but they were behind closed doors -- then within the first two weeks they get to feel that joy, that comfort, and then they pass away because of an overdose. We were just getting our feet wet...it was a lot of trial and error. A lot of the deaths did not stop for a good while until we kind of got some kind of semblance of harm reduction on site.

The theme of closed doors conveyed the dual valences of safety and risk at play in the SIP hotel initiative, especially in the early weeks prior to the addition of more robust harm reduction support and supplies. For both PEH previously residing outdoors or in congregate shelters, private hotel rooms with nursing and medical care on site afforded safety and dignity, yet for people who use drugs, the closed door created conditions where overdose could go unwitnessed with fatal outcomes. For the nurses working in SIP hotels, the combination of medical and psychosocial risk with threat of overdose death behind closed doors was both a traumatic and bonding experience. As one nurse explained, “it’s still emotional…the ability to help so many in a pandemic. We talk like its over but it’s not over… I’ve become very close to people on my team. We have other people who don’t understand what we are going through as you don’t want to traumatize other people. It’s overwhelming and special.”

## Discussion

Our study examined a housing intervention rapidly scaled up in the context of the COVID-19 pandemic. Medically supported SIP hotels had both individual and community impact: decreased acute care utilization, increased outpatient care engagement, and modified approaches to caring for people who use substances. Despite its strengths, our qualitative focus group results also expose the complexity of scaling up an emergency program among a vulnerable population, where newly housed PEH who used drugs died of overdose alone behind closed doors before improvements in harm reduction were instituted. Future efforts must be aware of and attempt to mitigate these harms. This study offers guidance on how to provide safe, equitable, and dignified temporary housing both in the setting of pandemic-like emergencies as well as in response to everyday disasters like prohibitive housing costs.

Our data suggest that there may be a role for a transitional intermediate-term housing like the SIP hotel program, particularly for PEH who may have spent decades living outside and have had minimal engagement with healthcare and social services due to medical racism and trauma. Some PEH may benefit from an initial intensive adjustment period similar to the SIP hotel model prior to obtaining permanent supportive or independent housing. The SIP hotels highlight the complexity of providing health services within a housing model for a population of both high and non-utilizers. The SFDPH approach to SIP placement, while first following a medical vulnerability scoring, also afforded prioritization of Black and transgender populations which have historically been systemically marginalized and subjected to significant medical trauma—particularly for PEH who may have spent decades living outside and have had minimal engagement with healthcare and social services due to past experiences of stigmatization, medical racism, and trauma [27]. Further research is needed to determine the effect of this type of intervention on the entrenched oppression built into healthcare and housing structures (Fig. 1).

The SIP hotel initiative spurred innovative developments in the provision of MOUD and in the triage of permanent supportive housing (PSH) within SF. In our study, many of the participants had Addiction Medicine specialty care visits with both specialists and generalists prescribing buprenorphine via novel delivery models. While the need is much greater to reduce continued morbidity and mortality among PEH who use drugs, such efforts to lower barriers can effectively empower people and disentangle them from the carceral system. The medical and nursing directors developed a list they referred to as “sick in SIP,” focused on residents with multiple chronic medical conditions who regularly engage with on-site nursing services. To date, 149 people were identified as “sick in SIP” and prioritized for PSH with access to on-site nursing.

### Limitations and Future Directions

Our study has several limitations. Our quantitative data does not fully capture care utilization within SF for PEH. We utilized SFDPH Epic data to quantify emergency visits and hospitalizations; while were able to view several local hospital systems (San Francisco General, UCSF, Kaiser, California Pacific/Sutter Health), our “high-cost system utilization” is likely an underestimate as our database is poorly synced with Dignity Health leading us to exclude services accessed at St. Francis Hospital in the Tenderloin neighborhood where majority of PEH reside. Further, the CCMS system tracks care utilization within SF starting in 2004, contributing to underestimation of lifetime of homelessness, incarceration, and service utilization outside of the city. We were further limited in by restricted access to methadone treatment data and other specialty SUD treatment data due to 42 Code of Federal Regulations (CFR) part 2 privacy laws [28]. Despite the socioeconomic demographics of our randomized stratified sample appearing slightly more vulnerable than other PEH in and outside of SIP, we assume that mental health and substance use data are vastly underreported within our sample due to extensive privacy laws as well as the low prevalence of community mental health providers visible via Epic. While non-fatal overdose was common within our cohort, many more PEH have experienced unintentional overdose than documented. We also are unable to capture the medical complexity of the numerous residents who opt out of care. Finally, our study only depicts care utilization patterns for the first 6 months after SIP limiting our conclusions on retention within outpatient lower-cost care models.

Future studies should focus on how the unique services offered during SIP housing (nursing stations, harm reduction supplies, meals, buprenorphine delivery) enhanced value(s) for PEH as the city of SF works to find PSH—that is truly supportive—for its most vulnerable citizens. In a parallel qualitative arm of our study, we aim to share the voices of SIP residents, clinicians, and CBOs from in-depth interviews to better illustrate the necessity and complexity of this example of temporary urban housing.

## Conclusion

The SF SIP hotel program was a social experiment affording highly serviced temporary housing to PEH during the COVID pandemic. The large-scale, rapid development of this program to provide housing, meals, and medical care to nearly 3000 PEH demonstrates the feasibility of rapid expansion of housing options when faced with the right combination of political, social, and medical pressures. Policymakers often turn to narrow, overly simplistic considerations of both impact and outcomes when evaluating programs such as the SIP hotel program—however, our data suggest social, medical, and economic value of temporary housing for PEH.

## Data Availability

The data that support the findings of this study are available on request from the corresponding author.
